# Effect of biological maturation on strength-related adaptations in young soccer players

**DOI:** 10.1371/journal.pone.0219355

**Published:** 2019-07-05

**Authors:** Iván Peña-González, Jaime Fernández-Fernández, Eduardo Cervelló, Manuel Moya-Ramón

**Affiliations:** 1 Department of Sports Science, Miguel Hernández University of Elche, Alicante, Spain; 2 Faculty of Physical Activity and Sport Sciences, University of León, León, Spain; Universidad Pablo de Olavide, SPAIN

## Abstract

Strength training is crucial for soccer players’ long-term development at early ages and the biological maturation may influence specific strength-training adaptations. The aim of this study was to propose a strength-training programme for the strength development of pre-pubertal players and to analyse the adaptations to this training programme in players with different maturity status. One hundred and thirty young male soccer players participated in an 8-week strength-training programme consisting of two sessions per week (20-minutes of a combination of plyometric and resistance exercises) which was conducted prior to their normal soccer training. Three maturity groups were defined according to the years from/to their peak height velocity (PHV) as Pre-, Mid- and Post-PHV. Initial differences between the maturity groups were found in anthropometrical (weight and height) and physical performance variables (One Repetition Maximum (RM), Peak Power output (PP), 30-m sprint and T-test). The strength-training programme was beneficial for the three maturity groups (p< 0.05) with general greater improvements for the Pre- and Mid-PHV groups, with *large* effects in RM, PP and T-test, than for the Post-PHV group (*moderate* effects). The strength-training programme proposed in the present study seems to be positive for the strength-related development in young soccer players especially for Pre- and Mid-PHV players. The differences in the training adaptations for players with different maturity status suggest the individualization of the training stimulus for the correct long-term development of the players.

## Introduction

Physical performance demands in soccer are related with maximal and explosive strength actions [1,2]. It is for this reason that strength training has become crucial in the players’ long-term development [3–5] and its evaluation can be used as a physical performance indicator in talent identification and selection processes at early ages [1]. Plyometric and resistance training methods have been reported as effective for the improvement of the strength-related actions in soccer, such as jumping, sprinting or changes of direction [5–7]. In this regard, plyometric training methodologies seem to achieve soccer-specific adaptations mainly by neuromuscular improvements [1,8] while resistance training is related with structural adaptations.

With the aim of creating a fair system to identify and select future talented soccer players at early ages, the structure of soccer has traditionally grouped young players into 1-year-categories. However, in the sports research field, there is an increasing interest in the idea that supports that inter-individual differences in biological maturation between players of the same category may influence the strength-related performance as well as the specific adaptations to the strength training [1,9].

Previous investigations reported greater adaptations to strength programmes, with plyometric and/or resistance training methodologies, for post-pubertal players and these support the idea of the “window of opportunity” of those players advanced in their maturation [3,7,9]. This period of accelerated gains in strength for post-pubertal players is supported by rapid gains in muscle mass due to an increment of androgen concentrations [10]. Nevertheless, contemporary long-term athlete development models have shown strength training to be important in pre-adolescence, highlighting a neural plasticity associated with pre-pubertal players that supports muscular strength development in these years through gains in neuromuscular adaptations as intra- and intermuscular coordination [10].

In the sports field, soccer trainers and coaches of pre-adolescent teams try to design strength programmes for the long-term development of their players. Controversy in the results of previous research about the adaptations to strength training between different maturity status groups of athletes leads to a lack of consensus about the strength training method in pre-pubertal period. Soccer specific adaptations to a plyometric training in combination with the normal soccer training showed greater gains in strength related abilities for more mature players [1], while school-aged populations have shown greater improvements in strength for pre- and post-pubertal players [5,7,9,11].

Taking the controversy about the maturity-related adaptation for a strength-training programme into account, this study aimed to propose a strength-training programme based on the contemporary long-term athlete development models for the strength improvement of pre-pubertal soccer players. The aim of the present study was to report the initial differences between young soccer players with different maturity status and also to analyse the physical performance adaptations according to their maturity status for a specific strength-training programme.

## Materials and methods

### Participants

One hundred and thirty young male soccer players from the first and second level of the Spanish regular league competition participated in the study. Mean ± standard deviation for the initial measurements of the sample are shown in Table 1. For the inclusion in the study, the participants must have attended a minimum of the 80% of the programme sessions. All players participated voluntarily in the study and their parents/guardians signed an informed consent. The study was approved (DPS.EC.01.17) by a Research Ethic Committee conforming to the recommendations of the Declaration of Helsinki.

**Table 1:** Initial measurement of players’ outcomes according to the maturity group (mean ± standard deviation).

	CG (n = 20)	Pre-PHV (n = 43)	Mid-PHV (n = 36)	Post-PHV (n = 31)
CA (years)	13.2 ± 1.1	12.8 ± 0.4	13.8 ± 0.6*^§^	14.6 ± 0.50*^§^ ^#^
Height (cm)	158.2 ± 11.1	154.9 ± 6.2	165.9 ± 4.8*^§^	171.9 ± 10.4*^§^ ^#^
Weight (kg)	50.5 ± 10.3	45.4 ± 5.7*	55.8 ± 5.0^§^	62.3 ± 6.6*^§^ ^#^
RM (kg)	66.1 ± 17.5	50.8 ± 12.4*	73.4 ± 17.1^§^	87.9 ± 15.4*^§^ ^#^
PP (W)	648.8 ± 235.1	544.2 ± 183.6	796.0 ± 239.7*^§^	951.6 ± 231.1*^§^ ^#^
30-m sprint (s)	5.0 ± 0.4	4.8 ± 0.2*	4.6 ± 0.2*^§^	4.5 ± 0.2*^§^
T-test (s)	9.3 ± 0.6	9.3 ± 0.5	8.7 ± 0.3*^§^	8.6 ± 0.4*^§^

CA: Chronological age; RM: One repetition maximum; PP: Peak power output;

*Statistically different from control group (CG)

^§^Statistically different from Pre-PHV

^#^Statistically different from Mid-PHV

### Procedure

A pre- post-test intervention design was carried out with an 8-week strength programme between pre- and post- testing sessions. As a result of the intervention, changes in physical performance of the young players between the pre- and post-test were assessed, and between-group analyses were carried out to compare the changes between maturity groups. All the participants were already familiarized with the physical performance tests and they had experience in the strength training. Each testing session was separated from the previous and following training session by at least 48 hours. During the intervention (8 weeks), players performed three normal training sessions (soccer-specific trainings in the field) plus two strength-training (ST) sessions per week. The normal soccer-specific trainings of the teams consisted in technical and tactical exercises designed by the coach and strength-related tasks were not allowed in these field sessions.

Players’ anthropometrical parameters as weight (kg), height (cm) and sitting height (cm) were initially evaluated. After the anthropometrical assessment, players performed a standardized warm-up, consisting in: (1) low-intensity running, (2) dynamic stretching and (3) the repetition of the squat movement with different loads. At the beginning, each player started with approximately the 50% of their weight and next series the load was incremented. Each player performed a minimum of three series of three to five repetitions and a maximum of five series previous to the recording one. The one repetition maximum strength (RM) and the peak power output (PP) in the half-squat exercise were estimated indirectly [12] using a Smith Machine (Technogym Trading, Gambettola, Italy) and recorded with a linear encoder (T-Force System, Ergotech, Murcia, Spain).

The time during a 30-m dash in a straight line and the agility T-test [13] were measured using photoelectric cells (Datalogic S6 Series, Bologna, Italy). Participants initiated the tests from a standing position 30 cm behind the photocell, which started a digital timer. Players performed two attempts with a 2-minute rest between trials, and the best of the two trials was used for analysis.

### Maturity status

Despite having some limitations and although new prediction equations are appearing in these last years, the assessment of the years from/to the peak height velocity (PHV) is the most commonly used indicator of the somatic maturation in the sports field [14]. The PHV indicates the theoretical benchmark of the maximum growth in height during adolescence which occurs, on average, around the age of 14 in males and 12 in females [15]. The prediction of the years from/to the PHV, also called “maturity offset”, gives us an accurate reference of the maturity status of the young athlete [16,17] and it is especially accurate in boys from 12 to 16 years old with an “on average” maturation [18].

For the later analysis, players were grouped into three maturity groups according to their years from/to PHV. Maturity groups were defined with the same name as in other studies for the easy understanding of the reader: Pre-PHV (n = 43; <0.5 years to PHV), Mid-PHV (n = 36; >0.5 years to and <0.5 years from PHV) and Post-PHV (n = 31; >0.5 years from PHV). Additionally, a control group (CG) was created with Pre-, Mid- and Post-PHV players (n = 20). The CG did not carry out the ST programme but participated in the normal soccer-specific trainings with their teams.

### Strength training programme

During the ST programme all players carried out three normal soccer-training sessions of 90 minutes of duration per week plus two extra ST sessions per week, consisting in 20-minutes of a combination of plyometric and resistance exercises (see Table 2), which were conducted prior to the normal soccer training. With the aim of designing a strength programme for the development of pre-pubertal players, the external load was small but the movement velocity required was maximal, looking for neuromuscular adaptations rather than structural ones. The CG performed the normal soccer training but not the extra ST work. Each training session was carried out at the same time of the day and one investigator supervised all ST sessions. Prior to each ST session, players carried out a standardised warm-up consisting in five minutes of running and three minutes of dynamic stretching. The ST programme was divided into two 4-week-long periods labelled Block 1 and Block 2. The aim of dividing the ST programme into two blocks was to increase the initial load of some exercises. Players were encouraged to perform the exercises at maximal velocity and to repeat the exercise as many times as possible in 30 seconds. The work/rest ratio was set as 1:1.

**Table 2:** Exercises and progression during the eight weeks of the ST.

	Block 1 (Weeks 1–4)	Block 2 (Weeks 5–8)
Semi-squat	*Without external load*	With a 10-kg disc
Lunge	*Without external load*	With a 5-kg ball
Side lateral lunge	*Without external load*	With a 5-kg ball
Single-leg squat (right)	*Without external load*	*Without external load*
Single-leg squat (left)	*Without external load*	*Without external load*
Dead lift	*Without external load*	With a 10-kg bar
Hip-thrust	*Without external load*	With a 5-kg ball
Multi-jumping	Two-legs jumping on an agility ladder	One-leg jumping on an agility ladder
Hip-rotation	With an elastic band	With an elastic band

### Statistical analysis

Data for the initial measurement of the players as well as for the pre- post-test analysis was reported as Mean ± SD. Differences in each group for physical performance tests were reported as the percentage of change, while the comparison in the pre- post-test changes between groups was reported with the effect sizes.

An initial one-way analysis of the variance (ANOVA) was carried out to compare the initial measurement between the three maturity groups and the CG, and a post-hoc analysis (Bonferroni) was used to report the initial statistical differences between groups. A paired-samples t-test was used for the analysis of the pre- post-test differences in each maturity group as well as in the CG for each physical performance test. The effect sizes of the between groups comparison about the training adaptations were analysed as trivial (< 0.19), small (0.20–0.49), moderate (0.50–0.79) and large (> 0.80) [19]. For the standardised effects of the ST programme, a magnitude-based inference analysis with a specific spreadsheet, “Compare two groups means” (www.sportsci.org/), was used. For this analysis, the smallest worthwhile difference in means in standardised units (Cohen’s d) was set at 0.2, representing the hypothetical smallest difference. The qualitative descriptor according to the chance of benefit was provided for the better understanding as possibly (25 to 75%); likely (75 to 95%); very likely (95 to 99.5%); most likely (>99.5%) [20].

All calculations were performed using Microsoft Excel (Microsoft, Seattle, Washington, USA) and SPSS Statistics (Statistical Package for the Social Sciences, Version 17.0), and the level of significance was set at p < .05.

## Results

The ANOVA with the post-hoc analysis showed initial differences between the different maturity groups (Pre- < Mid- < Post-PHV) as well as with the CG. The pairwise statistical differences between groups are shown in Table 1.

The statistical differences shown in the paired-samples t-test are reported in Table 3, as well as the effect size (95% CI) with the magnitude of the training effect, the descriptive probability of the benefit and the percentage of the change after the application of the ST programme. Significant differences for RM, PP and T-test were found between pre- and post-test evaluation for Pre-, Mid- and Post-PHV (*p<* 0.05) while no significant differences were found for the CG.

**Table 3:** Changes in physical performance for the different maturity status groups and control group (CG).

	PRE	POST	ES (95% CI)	Training effect	Probability of benefit	% of change
**RM (kg)**						
CG	66.1 ± 17.5	67.0 ± 17.4	-0.05 (-0.67; 0.57)	Trivial	*Likely*	4.9
Pre-PHV	50.8 ± 12.4	67.2 ± 11.2*	-1.39 (-1.83; -0.92)	Large	*Most likely*	32.3
Mid-PHV	73.4 ± 17.1	93.3 ± 18.5*	-1.12 (-1.60; -0.61)	Large	*Most likely*	27.1
Post-PHV	87.9 ± 15.4	100.8 ± 18.2*	-0.77 (-1.27; -0.24)	Moderate	*Most likely*	14.7
**PP (w)**						
CG	648.8 ± 235.1	719.1 ± 241.3	-0.30 (-0.91; 0.33)	Small	*Possibly*	14.6
Pre-PHV	544.2 ± 183.6	695.3 ± 162.1*	-0.87 (-1.29; -0.44)	Large	*Most likely*	27.8
Mid-PHV	796.0 ± 239.7	994.3 ± 224.1*	-0.85 (-1.33; -0.36)	Large	*Most likely*	24.9
Post-PHV	951.6 ± 231.1	1085.0 ± 243.8*	-0.56 (-1.06; -0.05)	Moderate	*Most likely*	14.0
**30-m sprint (s)**						
CG	5.1 ± 0.4	5.0 ± 0.6*	0.20 (-0.43; 0.81)	Small	*Possibly*	2.0
Pre-PHV	4.8 ± 0.2	4.8 ± 0.2	0.00 (-0.41; 0.41)	Trivial	*Possibly*	0.0
Mid-PHV	4.6 ± 0.2	4.5 ± 0.2*	0.50 (0.03; 0.96)	Moderate	*Very likely*	2.2
Post-PHV	4.5 ± 0.2	4.5 ± 0.2	0.00 (-0.50; 0.50)	Trivial	*Possibly*	0.0
**T-test (s)**						
CG	9.3 ± 0.6	9.1 ± 0.5	0.36 (-0.27; 0.98)	Small	*Possibly*	2.1
Pre-PHV	9.3 ± 0.5	8.9 ± 0.3*	0.97 (0.53; 1.39)	Large	*Most likely*	4.3
Mid-PHV	8.7 ± 0.3	8.4 ± 0.4*	0.85 (0.36; 1.32)	Large	*Most likely*	3.4
Post-PHV	8.6 ± 0.4	8.4 ± 0.4*	0.50 (-0.01; 1.00)	Moderate	*Very likely*	2.3

*(*p*<0.05): Significantly different from PRE

Table 4 shows the standardized pairwise comparison of the adaptations to the ST programme between groups. *Moderate* to *large* effects in the adaptations for RM and PP was shown in each maturity group when compared with the CG, and even these effects seem to be significant when CI does not cut cero. However, only *moderate* effects in the adaptations for the ST programme were shown in T-test between Pre-PHV and the CG without cutting cero. The rest of the pairwise effects with the CG were *trivial* and *small*. *Moderate* to *large* effects in the adaptations to the ST programme were found between the Pre- and Mid-PHV groups with the Post-PHV for the RM and PP tests, and the CI without cutting cero indicates the significant differences in the adaptations to the ST programme between these maturity groups.

**Table 4:** Pairwise comparison of the standardized effects in Cohen units (95% CI) for the changes between maturity groups.

	Control vs Pre-PHV	Control vs Mid-PHV	Control vs Post-PHV
RM	-1.51 (-2.08; -0.90)	-1.09 (-1.65; -0.49)	-1.01 (-1.59; -0.39)
PP	-0.54 (-1.08; 0.00)	-0.57 (-1.11; 0.00)	-0.15 (-0.71; 0.42)
30-m sprint	0.27 (-0.27; 0.80)	-0.07 (-0.62; 0.48)	0.23 (-0.35; 0.79)
T-test	-0.76 (-1.30; -0.20)	-0.33 (-0.88; 0.22)	-0.23 (-0.79; 0.34)
	Pre-PHV vs Mid-PHV	Pre-PHV vs Post-PHV	Mid-PHV vs Post-PHV
RM	0.18 (-0.27; 0.62)	0.92 (0.43;1.40)	0.60 (0.10; 1.09)
PP	0.18 (-0.26; 0.62)	0.51 (0.03; 0.98)	0.51 (0.01; 1.00)
30-m	-0.33 (-0.77; 0.12)	-0.07 (-0.53; 0.40)	0.28 (-0.21; 0.76)
T-test	0.43 (-0.03; 0.87)	0.54 (-0.06; 1.01)	0.11 (-0.38; 0.59)

## Discussion

The main results in this study suggest that a strength-training (ST) programme focused on neuromuscular adaptations (i.e. carried out at maximal voluntary velocity) rather than on the structural improvements (i.e. hypertrophy strategies) is beneficial for the strength development in youth soccer with generally greater improvements in Pre- and Mid-PHV groups. To our knowledge, it is the first reported specific ST programme for young soccer players that shows a general benefit in the adaptations for Pre- and Mid-PHV rather than Post-PHV players, and it could help professionals and coaches of pre-pubertal soccer players. A specific plyometric training programme for soccer players by Asadi et al., (2018) showed greater gains in jump and sprint performance for Post-PHV players. In addition, other references agree with the idea that Post-PHV have greater adaptations to the strength training, but these references were carried out without a soccer-specific population [7,9]. On the other hand, greater adaptations to a strength training programme for Pre-PHV boys was also shown in previous research [5,11] but also with school-aged population and not with soccer players. Anywise, the present study agrees with all these studies in the idea the idea of the importance of different physical performance and different training adaptations of young athletes with different maturity status in talent identification and selection processes [10].

Moreover, the improvements in the physical performance of young players are not only due to the prescription of the strength training but also to the natural development of young people [9]. In this regard, this study showed percentages of change from 2.0 to 14.6 in the different physical performance tests for the CG. These differences were *trivial* to *small* and with *possibly* to *likely* probability of benefit.

For the maximal and power strength evaluation (RM and PP), Pre- and Mid-PHV players had greater adaptations (24 to 32% of change with *large* effects) to the proposed ST programme than Post-PHV players (around 14% of change with *moderate* effects). Not only the magnitude of changes was reported but the standardized effects in the adaptation comparisons were also shown, with *moderate* to *large* effects in the Pre- vs Post-PHV and in the Mid- vs Post-PHV groups. Nevertheless, statistical analysis showed significant differences between pre- and post-test in all maturity groups. Thus, the proposed ST programme may be addressed to young soccer players from 12 to 15 years old to improve their maximal and explosive strength, highlighting the greater improvements of Pre-PHV players. Although the present ST programme was designed aiming to improve the physical performance of the Pre-PHV players in a greater extent, other possible reasons of the greater improvement of the Pre- than the Post-PHV in RM and PP may be the initial differences between these groups. In this regard, and in line with the results by Radnor et al., (2017), the proposed ST programme could be an insufficient strength stimulus to allow greater improvements for Post-PHV players. Post-PHV players may require, in function of their physical performance, more specific training stimulus [5].

Controversial results were found for the speed-related tests, as the 30-m sprint and the agility T-test. Although the relationship between the strength and speed improvements have been widely reported [21], the improvements in RM and PP shown in the present study were not joined by linear sprint improvements in the same maturity groups. Although some changes in pre- post-test analysis are statistically significant, *trivial* and *small* effects are shown for these changes. The changes shown in linear sprint in this study cannot support a clear benefit of the application of this ST programme. A possible explanation of this result is the high training stimulus of the current soccer-specific sample to speed-related tasks in their usual soccer training. In this regard, the present ST programme may be insufficient for the speed development of these young soccer players. On the other hand, each maturity group had similar adaptations after the proposed ST programme for the T-test than for the RM and PP (*large* effects (*p<* 0.05) in Pre- and Mid-PHV with *most likely* probability of benefit and *moderate* effects (*p<* 0.05) in Post-PHV with *very likely* probabilities of benefit). The controversy about the improvements in the agility T-test without clear improvements in linear sprint may be explained by the strength transference in agility tasks, the same as the change of direction, due to an efficient stretch-shortening cycle [7].

One of the main limitations of the present study was the control of the soccer-specific training loads. Players attended their usual soccer trainings with their usual team (with their chronological age peers) and differences between the soccer-specific training loads may appear. The authors tried to cushion this bias by blocking any kind of strength training during the soccer-specific training. In addition to this, the aggrupation of players by maturity status for the analysis usually appears in the bibliography as: Pre-PHV < -1 and Post-PHV > 1 in their maturity offset. The present study aimed to reduce the biological differences between groups to perform a more exhaustive analysis. However, due to the error associated with the maturity offset formula, the number of players classified into a wrong group may increase in comparison with other studies. The initial differences between groups, together with the small standard deviation in weight and height, indicated that the maturity groups were initially homogeneous, and thus the associated error in the PHV offset calculation [16] was relatively controlled.

## Conclusion

Given the relevance of strength in soccer, a strength-training programme focused on neuromuscular adaptations and carried out during eight weeks (twice per week) was shown in this study to be effective to improve strength-related physical performance in young soccer players, with generally greater improvements in Pre- and Mid-PHV players. The maturity-related physical performance differences, together with the differences in the adaptations to the strength training reported in this study, could be very useful for professionals in soccer in which it is very common that players with different maturity status practice and compete together. Individualized training stimulus for players with different maturity status may be successful in long-term soccer players’ development programs and may help soccer clubs in the identification and selection process of players at early ages.

## Supporting information

S1 File Sample. (XLSX)
